# Cognition at baseline and relationships 10 years after a first episode of schizophrenia^☆^

**DOI:** 10.1016/j.scog.2026.100433

**Published:** 2026-03-23

**Authors:** Anja Vaskinn, Anne-Kari Torgalsbøen

**Affiliations:** a Centre for Research and Education in Forensic Psychiatry, Oslo University Hospital, Oslo, Norway; b Department of Psychology, University of Oslo, Oslo, Norway

**Keywords:** Schizophrenia, Intimate relationships, Romantic relationships, Follow-up, Cognition, Social cognition

## Abstract

**Introduction:**

Social and nonsocial cognition are important predictors of functioning among people with schizophrenia, both cross-sectionally and longitudinally. It is unknown if this extends to functioning in close and intimate relationships. This study investigated if baseline cognition predicted relationship functioning ten years after a first episode of schizophrenia.

**Methods:**

Twenty-two participants with a first episode of schizophrenia underwent baseline assessment with MATRICS Consensus Cognitive Battery, providing scores for social and nonsocial cognition. Ten years later a clinical interview ascertained information about romantic relationship status and number of and frequency of contact with (talking, meeting) close friends. The predictive power of cognition for later relationships was examined with regression analyses.

**Results:**

One finding was statistically significant. Nonsocial cognition predicted how often someone talked to close friends ten years later.

**Conclusions:**

Baseline cognition, especially social cognition, had little impact on relationships ten years after a first episode of schizophrenia. Possible explanations are discussed.

## 1 Introduction

Schizophrenia is characterized by cognitive impairments (Schaefer et al., 2013; Savla et al., 2013; Green et al., 2019) with both social and nonsocial cognition providing strong input to the functional difficulties observed in individuals with the disorder (Halverson et al., 2019). The likely importance of social cognition for *social* functioning, is reflected in a commonly used definition of social cognition, i.e., “…the mental operations that underlie social interactions, including perceiving, interpreting and generating responses to the intentions, dispositions, and behaviors of others” (Green et al., 2008). It is conceivable that deficits in social cognitive domains such as emotional processing (Kohler et al., 2010) and theory of mind (Bora et al., 2009; Chung et al., 2014) can hinder the ability to establish and sustain close and intimate relationships, thereby contributing to the social difficulties encountered by individuals with schizophrenia (Murrihy et al., 2025). Cognition influences the opportunity to learn and to interact with others and could have long-term consequences for relationships. Studying this in first episode samples is especially suitable. If cognition impacts on later relationship functioning, targeted interventions early in the course of illness can be envisioned.

Individuals with schizophrenia express a desire for sexual (Huguelet et al., 2015; de Boer et al., 2015) and romantic (de Boer et al., 2015) connections and a wish to be intimate (McCann et al., 2019) but may face challenges in establishing and maintaining such relationships (Barker and Vigod, 2020). Social difficulties are seen in both close and intimate, as well as more distal, social relationships. People with schizophrenia tend to have few friends, 1–2, although the quality of the friendship is good (Harley et al., 2012). Whereas some are happy with the number of friends they have, the majority report that they would like to have more friends, in turn linked to lower quality of life (Tee et al., 2022). Schizophrenia is also associated with a high risk of living alone (Hakulinen et al., 2019), though cultural differences are likely, as marriage rates among persons with severe mental illness are high in Asian countries and low in Scandinavian countries (Lyngdoh et al., 2023).

With the backdrop that the functional difficulties in schizophrenia extend to close and romantic relationships, and that social and nonsocial cognition predict functioning, the aim of this study was to explore these associations. In this longitudinal study of first episode schizophrenia (FES), we examined whether social and nonsocial cognition at baseline predict friendships and romantic functioning after ten years. We hypothesized that social cognition would be a stronger predictor than nonsocial cognition, and that the association would be significant.

## 2 Materials and methods

### 2.1 Participants

Thirty-one individuals with FES were referred to the Oslo Schizophrenia Recovery (OSR) study over a period of four years (2007–2011). They were recruited from various mental health service institutions in the Oslo area of Norway, the majority from units specializing in early intervention and treatment of psychosis. Twenty-eight fulfilled the following inclusion criteria: age ≥ 18 years, first episode of mental illness within the spectrum of schizophrenia and psychosis according to DSM-IV (American Psychiatric Association, 1994). First episode was defined as referral to OSR within five months of their first contact with mental health service institutions. Exclusion criteria were a diagnosis of affective disorder, IQ < 70, and presence of head trauma. All participants could read and write Norwegian fluently. The complete sample represented about 60% of the incidence cases from the catchment area and is thus considered representative for the FES group.

### 2.2 Cognitive measures

Social and nonsocial cognition was measured with the MATRICS Consensus Cognitive Battery (MCCB; Nuechterlein and Green, 2009). We used a composite nonsocial cognitive T-score, based on the six nonsocial MCCB domains (speed of processing, attention/vigilance, working memory, verbal learning, visual learning, reasoning/problem solving). Social cognition was assessed with the MSCEIT Managing Emotions subtest, a test of emotional processing. Eight short vignettes are read aloud to the test-taker. The vignettes describe challenging social situations. Four possible actions are presented after each vignette, and the test-taker is instructed to rate how effective or ineffective each action is, using a five-point scale. We used T-scores for MCCB social and nonsocial cognition in the statistical analyses.

### 2.3 Romance and relations

Romantic relationship status was indexed by a dichotomous categorical variable, i.e., having or not having a boyfriend or girlfriend at 10-year follow-up. Marriage or cohabiting counted as having a boyfriend or girlfriend. Three variables measured close relationships: number of close friends (continuous variable), frequency of talking with close friends during a week, including texting, talking on the phone, and messaging online (categorical ordinal variable), and frequency of meeting close friends during a week (categorical ordinal variable). This information was collected as part of a clinical interview.

### 2.4 Other measures

Symptom level was measured with the Positive and Negative Syndrome Scale (PANSS: Kay et al., 1987). We report the scale scores for positive, negative and general symptoms. We assessed functioning using the Global and Social Role Functioning Scale (Cornblatt et al., 2007). In previous OSR papers, cognition predicted short (Torgalsbøen et al., 2014) and long-term (Fu et al., 2017) functioning assessed with this instrument, but in the current paper global social and role functioning did not constitute study variables and hence, are only reported.

### 2.5 Statistical analyses

We performed four separate regression analyses for the four different 10-year relational outcomes. Independent predictors in all regression analyses were baseline social (MSCEIT subtest) and nonsocial (other MCCB subtests) cognition. Their predictive value for romantic relationship status was examined with a binary logistic regression, whereas a multiple linear regression analysis was used to predict number of close friends. Cognitive predictors of frequency of contact with close friends (talking or meeting) were examined with two multinomial logistic regressions. In the logistic regressions, the two cognitive predictors were entered hierarchically in separate steps, using forced entry. Nonsocial cognition was entered in the first block, social cognition in the second. In the multiple linear regression, they were entered in the same step.

There was no multicollinearity. The Box-Tidwell procedure showed linear relationships between the predictors and the logit of the categorical dependent variable in the binary logistic regression analysis. Assumption testing for the linear regression analysis provided plots indicative of homoscedastic and normally distributed data. Analyses were undertaken in SPSS version 30.0.

## 3 Results

Table 1, Table 2 show baseline and 10-year follow-up data. At 10-year follow-up, 22 participants (79%) were retained and included in the current study. Of these, 29% were in a close relationship, similar to other first episode samples (Bertelsen et al., 2008), and 18% had children at 10-year follow-up.

**Table 1:** Demographic and clinical characteristics of individuals with first-episode schizophrenia at baseline and at 10-year follow up (*n* = 22).

	Baseline Mean (SD) Min-max	10-year follow-up Mean (SD) Min-max
Demographics		
Age	21.1 (2.7)	31.6 (2.7)
Women/men (n)	10/12	10/12
Education level n (%)		
Elementary school	8 (36.4%)	6 (27.3%)
High school	7 (31.8%)	8 (36.4%)
Some college	6 (27.3%)	4 (18.2%)
Bachelor degree or more	1 (4.5%)	4 (18.2%)
Clinical information		
PANSS		
Positive symptoms	18.3 (5.4)	8.1 (2.3)
Range 7–49	8–30	7–16
Negative symptoms	20.7 (4.3)	8.6 (2.1)
Range 7–49	13–31	7–13
General symptoms	38.1 (8.9)	19.9 (3.9)
Range 16–112	22–54	16–30
Inpatient/outpatient (n)	14/8	0/13⁎
Functioning: Global and Social Role Functioning Scale		
Social functioning	6.2 (0.9)	7.1 (1.2)
Range 1–10	3–8	4–9
Role functioning	4.1 (1.9)	6.9 (1.4)
Range 1–10	2–7	4–8

PANSS = Positive and Negative Syndrome Scale.

⁎ *n* = 9 participants no longer received any treatment at 10-year follow-up.

**Table 2:** Romance and relations among individuals with first-episode schizophrenia at baseline and at 10-year follow up (n = 22).

	Baseline	10-year follow-up
Boyfriend/girlfriend (yes/no)	2/20	8/14
Number of close friends	2.5 (1.24)	2.1 (1.35)
Frequency of talking with friends	(n = 16) a	(n = 21) b Never: 3
	0–1 times per week: 9	Once a week: 9
	Every other day: 2	Every other day: 4
	Daily: 5 a	Daily: 5
Frequency of meeting with friends	(n = 21) b	
-never	-5	−9
-1-2 times a week	−10	−10
-3 times or more	−4	−2

a *n* = 16. This question was added to the questionnaire some time after study start-up. It is therefore missing for the first participants in the study.

b *n* = 21 due to missing data.

See Table 3 for the regression results. Neither social nor nonsocial cognition predicted romantic relationship status. The overall binary logistic regression model was non-significant.

**Table 3:** Regression analyses for the prediction of relationships at 10-year follow-up among individuals with first-episode schizophrenia at (n = 22).

		β	F	df	*p*-value	R^2^	Adjusted R^2^
Number of close friends			2.54	2, 19	0.154	0.21	0.13
	Nonsocial cognition	0.374			0.107		
	Social cognition	0.157			0.486		
			**χ**^**2**^			**Cox and Snell R**^**2**^	**Nagelkerke R**^**2**^
Romantic relationship status			1.36	2	0.506		
Frequency of talking with friends			20.00	6	**0.003**	0.614	0.664
	Nonsocial cognition		13.73	3	**0.003**		
	Social cognition		4.43	3	0.218		
Frequency of meeting with friends			5.95	4	0.203	0.247	0.291
	Nonsocial cognition		4.85	2	0.088		
	Social cognition		3.17	2	0.205		

Significant results appear in bold font.

Cognition at baseline did not predict number of close friends at 10-year follow-up. The multiple linear regression analysis was non-significant. Cognition explained 13% of the variance, but neither social nor baseline cognition were significant predictors.

Baseline cognition predicted frequency of talking with close friends at 10-year follow-up. The overall multinomial logistic regression model was significant, explaining between 61% and 66% of the variance. Nonsocial cognition made a significant unique contribution. Social cognition did not. Nonsocial cognition remained a significant predictor (χ^2^_(3)_ = 10.39, *p* = 0.016) in a new model (χ^2^_(6)_ = 17.68, *p* = 0.006) that controlled for baseline Social Role Functioning, which did not have a significant contribution (χ^2^_(3)_ = 2.12, *p* = 0.548).

Frequency of meeting close friends was not predicted by baseline cognition, with the multinomial logistic regression providing a non-significant overall model.

## 4 Discussion

In this study, we examined if baseline cognition predicted friendships and romantic relations in individuals with a first episode of schizophrenia, 10 years later. We found little support for our hypotheses. The analyses provided only one significant association: nonsocial cognition predicted frequency of talking with friends at ten-year follow-up. Social cognition at baseline was not associated with any outcome measure, in contrast to our hypothesis that social cognition would be not only a significant predictor, but stronger than nonsocial cognition.

We can think of several reasons why cognition turned out to be a weak predictor of intimate relationships after ten years. Obvious reasons are the sample size and restricted statistical power, and the time passed. Ten years is a long time, especially in young people's lives, with experiences and development mutually shaping and influencing everyone's life course. Perhaps cognition at baseline is too distal, and factors closer in time play a more important role for intimate relationships after ten years. This would be in line with a study by McCleery et al. (2016), where the predictive power of social cognition for functional outcomes diminished with increasing follow-up periods. It could also be that other baseline features of schizophrenia have more predictive power than cognition. The only significant difference at baseline was higher levels of positive (*p* = 0.008) and general (*p* = 0.023) symptoms among those lost to follow-up than in the current sample (Torgalsbøen et al., 2025). We cannot dismiss the possibility that symptoms play a role for long-term outcome, as the individuals with most severe symptoms did not participate at 10-year follow-up.

Our study yielded one significant finding, however. How often someone talked with friends at follow-up, was predicted by nonsocial cognition ten years earlier. This aligns with studies that have found cognition to predict long-term functioning up to 20 years later (Kharawala et al., 2022). Perhaps frequency of talking with friends is more of a proxy for real-world functioning as assessed in other studies, such as activities, occupations, hobbies, and interests involving active social participation. A speculation is that our three other measures tap into slightly different and more intimate aspect of relationships, and that more intimate relations have other underpinnings. Another possibility is that the use of more nuanced measures of relationship functioning, with a setup more like established measures of social functioning, going beyond categorical or dichotomous variables, will provide other results.

We found no support for the hypothesis that social cognition would predict long-term outcome. Social cognition has not had a strong influence on outcome in previous investigations from OSR, although a moderate-sized difference in social cognition was found between remitted and non-remitted participants after six months (Torgalsbøen et al., 2014). In our study, social cognition is assessed with one test, MSCEIT Managing Emotions, a measure of emotional regulation (Mayer et al., 2003). It was included in the MCCB because its psychometric properties were known (in healthy samples), but with only one study undertaken among individuals with schizophrenia (Nuechterlein et al., 2008). An early study of its psychometric properties in a schizophrenia sample (Eack et al., 2010) published not long after the launch of the MCCB, gave reason for concern, as validity estimates were not satisfactory. The strongest association was found with a neurocognitive composite score (discriminant validity), and correlations with clinician-rated social cognition (convergent validity) were modest and non-significant (Eack et al., 2010). Although it has qualities – it has for instance been found to uniquely predict quality of life, beyond the impact of the nonsocial tests in the MCCB (DeTore et al., 2018) – it is unclear how well it operationalizes social cognition, when administered alone. We agree with an early recommendation (Eack et al., 2010) that social cognitive assessment should be complemented with tests that tap into other social cognitive domains. In this specific instance, we believe this is especially important. Managing emotions may be less relevant for intimate relationships than recognizing and understanding social and emotional cues and the intentions of others. Given the wish of persons with schizophrenia to be intimate (McCann et al., 2019), future studies should investigate how difficulties in these social cognitive domains may relate to close relationships.

Overall, baseline cognition in first-episode schizophrenia was not a strong predictor of friendships and romantic relationships ten years later. Preliminary evidence does suggest, however, that nonsocial cognition may impact on frequency of talking with friends. Future research using larger samples and more fine-tuned measures are encouraged.

## CRediT authorship contribution statement

**Anja Vaskinn:** Writing – review & editing, Writing – original draft, Investigation, Formal analysis, Conceptualization. **Anne-Kari Torgalsbøen:** Writing – review & editing, Writing – original draft, Project administration, Methodology, Investigation, Data curation, Conceptualization.

## Ethical approval

After carefully describing the study and the procedures involved, the principal investigator (author AKT) obtained written informed consent from all participants. The study was approved by Norway's Regional Ethics Committee for Medical and Health Research (South East) (2007/1139) and conducted according to the Helsinki declaration.

## Funding

This research did not receive any specific grant from funding agencies in the public, commercial or non-profit sector.

## Declaration of competing interest

The authors declare that they have no known competing financial interests or personal relationships that could have appeared to influence the work reported in this paper.

## Data Availability

The participants of this study did not give written consent for their data to be shared publicly. Due to the sensitive nature of the research, supporting data is not available.
